# A novel decoding strategy for ProteinMPNN to design with less visibility to cytotoxic T-lymphocytes

**DOI:** 10.1016/j.csbj.2025.07.055

**Published:** 2025-08-13

**Authors:** Hans-Christof Gasser, Ajitha Rajan, Javier A. Alfaro

**Affiliations:** a School of Informatics, University of Edinburgh, UK; b Riddell Centre for Cancer Immunotherapy, Arnie Charbonneau Cancer Research Centre, University of Calgary, Canada; c Department of Biochemistry and Molecular Biology, Cumming School of Medicine, University of Calgary, Canada

**Keywords:** Protein deimmunization, MHC Class I, Protein design, ProteinMPNN, Decoding strategy

## Abstract

Due to their versatility and diverse production methods, proteins have attracted a lot of interest for industrial as well as therapeutic applications. Designing new therapeutics requires careful consideration of immune responses, particularly the cytotoxic T-lymphocyte (CTL) reaction to intra-cellular proteins. In this study, we introduce CAPE-Beam, a novel decoding strategy for the established ProteinMPNN protein design model. Our approach minimizes CTL immunogenicity risk by limiting designs to only consist of kmers that are either predicted not to be presented to CTLs or are subject to central tolerance that prevents CTLs from attacking self-peptides. We compare CAPE-Beam to the standard way of sampling from ProteinMPNN and the state of the art (SOTA) technique CAPE-MPNN. We find that our novel decoding strategy can produce structurally similar proteins while incorporating more human like kmers. This significantly lowers CTL immunogenicity risk in precision medicine, and represents a key step towards reducing this risk in protein therapeutics targeting a wider patient population.

Source: https://github.com/hcgasser/CAPE_Beam.

## Graphical abstract

**Figure:**
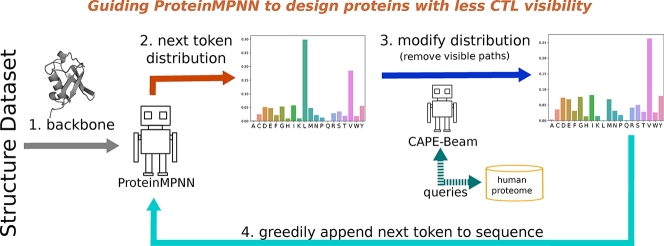

## 1 Introduction

Fueled by advances in AI methods, the field of computational protein design (CPD) has experienced much growth and attention recently. Protein design foundation models like ProteinMPNN [1] are being used to predict a protein's amino acid (AA) sequence based on its desired 3D structure. However, none of these models account for immune-system reaction to the designed sequences – which can be a crucial consideration in therapeutics. In particular, as AI-designed intra-cellular protein therapeutics incorporate novel functions and diverge from native sequences, they may be increasingly recognized by CTLs, potentially reducing efficacy or triggering autoimmune responses, as seen in cases of anti-transgene immunity [2], [3]. In this work, we will use the terms peptide and kmer interchangeably. In reality, a kmer is a short, continuous subsequence of a protein's AA sequence of length k, while the peptide is the actual molecule that has this sequence.

Kmers that are **(1)** not presented on the cell surface (**hidden**) or **(2)** can be found in the human proteome (**self kmers**) should not result in a CTL response. CTLs recognize their targets based on short protein derived peptides (mostly 8-10mers [4, Section 4-15]) that are presented on the cell's surface via the MHC Class I (MHC-I) pathway (see Section 2.1). To prevent auto-immunity, CTLs are trained in the thymus to tolerate peptides that originated from human proteins.

It is worth noting that the MHC-I system presents only a fraction of all potential kmers to the extracellular environment [5]. So, based on insight (1) and this fact, immunogenicity could be avoided by restricting the designed proteins to only include those hidden kmers. We believe that this approach will only be workable for precision medicine solutions that focus on a single patient. Based on NetMHCpan-4.1 [6] predictions with a rank cutoff of 2%, we estimate that approximately 10% of uniformly sampled 8-10mers are predicted presented in a single individual. However, this number increases significantly to cover a substantial part of the population. Designing complex protein structures excluding a large proportion of all potential kmers is a challenging task.

Another approach would be to leverage insight (2) by restricting protein designs to only include kmers found in the human proteome. We observe that although 8-10mers in the human proteome represent only a minuscule fraction of the entire spectrum of possible kmers (less than 0.02%), they nevertheless give rise to the entire complexity of human biology. This observation supports the hypothesis that a diverse array of structures can be assembled exclusively from self kmers. CTLs should be tolerant to all peptides derived from these. Except for some variation in the human genome, the resulting drugs would also not have to be personalized. In general, making the protein sequence look more similar to human proteins seems to reduce immune-reactions. This is highlighted by the fact that T-cell receptors (TCRs) bind far less strongly to tumor-associated antigenic peptides – which have sequences very similar to those found in the human proteome, than to viral ones [7]. In addition, Richman et al. [8] found that cancer neoantigen dissimilarity to the self-proteome predicts immunogenicity. Also, Rolland et al. [9] established a connection between higher kmer similarity in HIV proteins and reduced immunogenicity.

We introduce CAPE-Beam, a novel decoding strategy for ProteinMPNN that allows controlled incorporation of kmers from the human genome into generated protein designs. Specifically, CAPE-Beam enables designs to include either **self kmers** (those present in the human genome) or **hidden** kmers (those not presented on the cell surface). Our experiments show that restricting the design exclusively to self kmers leads to very low predicted TM-scores. In contrast, allowing only self 6-mers, while permitting longer kmers to be non-self but hidden, yields significantly higher predicted TM-scores in many cases.

## 2 Background

We now briefly discuss the detection of foreign intra-cellular proteins by the adaptive immune-system as well as two key machine learning (ML) elements for this paper. The first is ProteinMPNN – the foundation model used by our decoding strategy. The second are some basic decoding strategies for auto-regressive (AR) models like ProteinMPNN.

### 2.1 Detection of intra-cellular proteins

Their diverse and flexible designs allow proteins to fulfill a wide range of functions in biology. Proteins consist of chains of AAs. The distinct biochemical properties of AAs cause these chains to fold into different 3D structures. Within a cell, proteins are constantly being produced and degraded – leaving short AA sub-chains (peptides/kmers) behind. The MHC-I pathway presents a subset of these (mostly about 8-10 AAs long) on the cell surface. Which ones will depend on the MHC-I alleles expressed by the individual. The peptides displayed in this manner can subsequently be monitored by CTLs. These undergo training in the thymus to become tolerant of peptides inherent to the human proteome, thereby inhibiting auto-immunity. Conversely, foreign proteins are capable of inducing destruction through CTL under appropriate conditions [4].

### 2.2 ProteinMPNN

ProteinMPNN [1] is a protein design model that generates AA sequences conditioned on a desired 3D protein-backbone structure (the template), using an AR approach. With only around 1.7 million parameters, it is a relatively compact model. Notably, it also supports the design of protein complexes composed of multiple AA chains.

ProteinMPNN consists of an *Encoder* and a *Decoder*. The *Encoder* processes the spatial input structure and encodes it into a contextual representation. This is then used by the *Decoder* to iteratively generate the sequence. The decoding order can be freely specified, enabling the user to fix certain parts of the sequence in advance.

This paper explores an alternative approach to sample from the AA distribution produced by the model at each decoding step.

### 2.3 Decoding strategies

An AR language model typically outputs a set of logits – unnormalized log-probabilities – for all possible next tokens or categories. The simplest decoding strategy is greedy decoding, where always the token with the highest logit score is selected. Alternatively, one can apply a softmax function to convert the logits into a probability distribution, optionally scaled by a temperature parameter. A temperature of one leaves the distribution unchanged. As the temperature is lowered and converges to zero, the distribution sharpens and the decoding strategy approaches greedy decoding. In contrast, increasing the temperature will cause the distribution to flatten towards a uniform distribution – increasing randomness. The probabilities produced by the softmax function can then be used to sample the next token stochastically. Variants of this are *top-k* sampling, in which only the most probable *k* next tokens are considered for sampling. Or, *top-p* sampling, which only takes into account the smallest set of tokens that has a cumulative probability of more than *p*.

A fundamentally different approach is *beam search*. One of its earliest implementations is attributed to Lowerre [10]. In *beam search*, *k* partial sequence generations (beams) are tracked in parallel. At each step, every beam is expanded with all possible next tokens, resulting in k×V candidate continuations (where *V* is the vocabulary size). The overall *k* most likely candidates are retained for the next step, and the process repeats. Based on this concept, we developed the CAPE-Beam decoding strategy. For a broader overview and comparison of decoding strategies in the context of large language models (LLMs) see Shi et al. [11].

## 3 Related work

The problem to be addressed by computational protein design (CPD) is only loosely defined. Often times, the inverse folding problem is meant - that is, finding an AA sequence that folds into a given 3D structure. However, the term is also used more broadly to describe the generation of an AA sequence based on other constraints.

In the context of the inverse folding problem, traditional physics-based approaches aim to identify AA sequences that minimize a scoring function for a given confirmation. These scoring functions typically include approximations of the molecule's potential energy, but often also incorporate statistical terms to favor naturally looking structures [12]. The well-known *Rosetta Packer* [13], [14] for example uses Monte Carlo simulations to search for energetically favorable AA sequences. Yachnin et al. [15] extended this method by adding an additional term to the Rosetta score function that penalizes peptides likely to be presented on MHC Class II (MHC-II). Thereby they seek to reduce visibility to helper thymus dependent lymphocytes (T-cells) and as a consequence prevent an antibody (Ab) response. This idea was further adapted in the development of CAPE-Packer [16], which introduces a similar additional scoring term aimed at reducing visibility to CTLs via the MHC-I presentation pathway.

The recent advances in ML approaches have reinvigorated the field of CPD. Instead of minimizing a score function, these methods learn the sequence-structure relationship directly from data. Typically, a diffusion model like RFdiffusion [17] or FoldingDiff [18] is used to generate a 3D protein structure. The inverse-folding task of finding a corresponding AA sequence is then handled by models like ProteinMPNN [1] or ESM-IF [19].

Most work that used or adapted these generative models for immunogenicity concerns has focused on reducing Ab responses. For example, Lyu et al. [20] employed a Variational Autoencoder (VAE) approach to design novel adenovirus capsid proteins to evade vector detection by pre-existing Abs. Similarly, Bootwala et al. [21] applied the protein design framework of Zhou et al. [22] and the inverse folding graph neural network (GNN) model developed by Ingraham et al. [23] to re-surface the L-asparaginase protein to avoid Ab detection.

In contrast, our recent work has focused on minimizing CTL responses. We introduced CAPE-XVAE [16], a VAE based model, and CAPE-MPNN [24], a fine-tuned version of ProteinMPNN [1], specifically to reduce the number of MHC-I presented kmers in designs for a single MHC-I genotype. While our previous work did not take advantage of central tolerance, this work introduces a novel decoding strategy that explicitly prefers sampling self kmers.

## 4 Methods

This section first outlines our method for assessing peptide presentation via the MHC-I pathway. We then introduce the CAPE-Beam decoding strategy. Finally, the evaluation procedures used to assess the sequences generated by CAPE-Beam are described.

### 4.1 Predicting presented peptides

One of the most commonly used tools for predicting peptide presentation via the MHC-I pathway is NetMHCpan-4.1 [6]. Given a peptide and a specific MHC-I allele it returns a score indicating the probability of cell surface presentation. Based on this a rank is calculated which compares the peptide's score to the one of random natural peptides. A rank below 2% includes “weak binders”, while the stricter threshold of 0.5% corresponds to “strong binders” [6].

To identify presented peptides during the evaluation of our designed proteins, we directly use NetMHCpan-4.1 and classify any 8-10mer with a rank value below 2% as presented. However, because NetMHCpan is computationally intensive – particularly during sequence design, where many predictions are needed, we constructed a fast, approximate classifier based on position weight matrices (PWMs). Further details on this method can be found in Appendix A as well as in [24]. In contrast to [24] here we are generating a PWM for each MHC-I allele and peptide length (8-10) separately. It is important to note that this PWM based classifier is used only during sequence design. For evaluation, all reported results and figures are based on NetMHCpan-4.1 predictions of peptide presentation, using a rank threshold of 2%.

### 4.2 CAPE-Beam decoding strategy

Our method is a novel decoding strategy for ProteinMPNN, designed to ensure that all kmers in the generated protein sequence are either (1) part of the human proteome – in which case central tolerance should prevent an immune-reaction by CTLs – or (2) not presented on the cell surface at all.

**Fig. 1:**
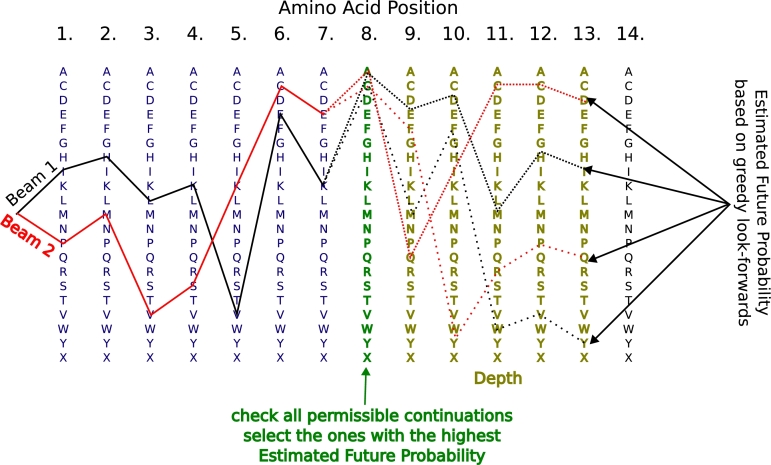
**Protein sampling:** AAs are added to the protein sequence one after the other. At each newly sampled position (green), all possible extensions are checked for permissibility. For each permissible extensions, a single permissible forward-looking continuation (illustrated as dashed lines) is greedily sampled for up to depth steps to estimate its *future probability*. For every beam (e.g., Beam 1 in black and Beam 2 in red), there will be up to 21 of those. The width candidate extensions with the highest estimated future probability are retained and expanded in the next step. This process continues iteratively until all residues have been determined.

Inspired by the classic beam search (see Subsection 2.3), our method generates width sequence beams in parallel (see Fig. 1). In accordance with the standard procedure in ProteinMPNN, we first sample the unresolved (missing coordinates) positions of the input template structure. Afterwards, we sample the remaining positions in an increasing order.

To extend a beam by one token, there are 21 (all standard AAs plus ‘unknown’) candidate extensions. To determine whether an extension is permissible, we check that all newly formed kmers – up to a length of max_checked_kmer_len – are permissible. For example with a max_checked_kmer_len of 4, extending the sequence beam IHLKVEK with a candidate token  leads to the following new kmers: , K, EK and VEK (see Fig. 1, Beam 1). A kmer is considered permissible if:

• **For lengths** *k*≤ **min_self_kmer_len:** the kmer must appear in the human reference proteome (GRCh38, Ensembl v94).
• **For lengths min_self_kmer_len** <*k*≤ **max_checked_kmer_len:** the kmer can either be a self kmer or be predicted not to be presented by the MHC-I pathway (see Subsection 4.1). These hidden non-self kmers are allowed, but penalized. Specifically, the candidate extension's estimated *future probability* (see below) is scaled by a factor of non_self_prob_factor^depth^. This biases the model towards using self kmers rather than relying on predicted non-presentation.

At each step, up to width × 21 candidate extensions may be permissible. The width candidates with the highest *future probability* are retained for the next iteration. In the special case that no candidates remain, the algorithm will select any width ones - however, this is in reality only the case if we do not allow for hidden non-self kmers. When all residues are decoded, the beam with the highest sum of all individual AA log-probabilities is selected as the final design.

The *future probability* mentioned above is estimated by greedily extending each candidate extension for an additional depth steps, using only permissible continuations. At each step, the log-probabilities of the most likely permissible AAs—produced by ProteinMPNN—are aggregated to compute the overall estimate.

To prevent beam collapse (i.e., beams being different initially but then converging on identical suffixes), we group all beams that have the same last 5 AAs, only keeping the most likely one of each group. A list of the CAPE-Beam decoding parameters can be found in Table 1.

**Table 1:** **CAPE-Beam decoding parameters:** For testing we disregarded forcing self-kmers longer than 6mers and only considered a non_self_prob_factor of 0.9.

Name	Values (validation)	Values (testing)
max_checked_kmer_len	10	10
min_self_kmer_len	5,6,7	5, 6
non_self_prob_factor	0., 0.1, 0.5, 0.9, 0.99	0.9
depth	two times min_self_kmer_len	two times min_self_kmer_len
width	10	10

### 4.3 Template dataset split

We adopted the same methodology for creating the dataset splits as was used for the *Specific Proteins* in Gasser et al. [24]. This ensures that the test templates were not used during ProteinMPNN training or validation, and the validation templates were not used during ProteinMPNN training. We expanded the validation set to 15 proteins and the test set to 20. The full list of protein templates used in each dataset split is provided in Table 2.

**Table 2:** **Template dataset splits:** The PDB IDs of the protein templates included in each dataset split.

Validation	1B9K, 1OA4, 1QTS, 1QWK, 1TEJ, 1TJE, 1UIZ, 1XGD,
	2C2X, 2QT4, 2R90, 4BVK, 4RQG, 5XBH, 6Q3V

Test	1A2O, 1A3H, 1P3C, 1PGS, 1QKD, 1S5T, 1X0M, 2BK8,
	3O6A, 3RFW, 3SFT, 3TIP, 3WOY, 4BOK, 4QTZ, 5OA9,
	5TPJ, 5V5F, 6PNW, 6TPT

### 4.4 Evaluation

We compared CAPE-Beam designs against three benchmark sources: (1) the original template proteins, (2) sequences generated by the standard ProteinMPNN decoding strategy (using a temperature of 0.1), and (3) designs produced by CAPE-MPNN using the method described in [24] (temperature of 0.1, checkpoint 458340e4:epoch_20). As ProteinMPNN checkpoint, we always used v_48_020. Our comparisons then evaluate structural similarity to the original templates, the presence and nature of potentially immunogenic kmers, and various predicted molecular properties.

#### 4.4.1 Structural similarity

To assess the structural similarity between our designs and the original protein templates, we first used ColabFold to predict 3D structures for our generated AA sequences. ColabFold is based on the well-known structure prediction tool AlphaFold-2 [25], [26], [27]. Structural comparisons were then performed using the TM-score, a widely used metric for evaluating the similarity of protein structures [28]. Scores below 0.2 are associated with random, unrelated proteins. Scores above 0.5 point to the same fold in SCOP/CATH.^2^

#### 4.4.2 Potentially immunogenic kmers

To evaluate the immunogenic potential of a protein sequence, we examined each of its 8-10mers and determined whether it belongs to the human proteome and if it is predicted to be presented on the cell surface.

As described in Section 4.1, presentation was assessed using NetMHCpan-4.1 [6] applying a 2% rank threshold. A peptide was considered presented if its predicted rank fell below this threshold for at least one of the six MHC-I alleles in the hypothetical patient's genome.

We report the fraction of presented non-self 8-10mers, i.e., the number of non-self 8-10mers that are presented by any allele divided by the total number of 8-10mers. In principle, CAPE-Beam should eliminate such kmers entirely. However, due to the discrepancies between the predictors used during design and evaluation (see Section 4.1), a small number may still arise.

Inspired by work suggesting a link between the similarity of a peptide to the self-proteome and its immunogenicity [7], [8], [9], we examine the resemblance of non-self 8-10mers incorporated into the sequences to those in the human proteome. For each non-self kmer we calculate our “BLOSUM62 dissimilarity” by first identifying the kmer in the human proteome that has the highest BLOSUM62 alignment score to the non-self kmer (without indels). Then we subtract this number from the BLOSUM62 alignment score [29] of the non-self kmer with itself.

#### 4.4.3 Molecular properties

To predict some of the designs' molecular properties, we used DE-STRESS [30]. It analyzes designs using SOTA predictors for various kinds of molecular properties. Our analysis focuses on the following metrics:

• *Rosetta score per AA:* A common assumption in protein design is that a protein will spend most of its time in low energy regions of its conformational space. The well known *Rosetta* suite of tools offers a score function that combines physical energy as well as statistical terms (see [12]). Therefore it uses Rosetta Energy Units (REUs) instead of standard energy units. To allow comparisons across proteins of varying lengths, we normalize by the number of AAs in the protein. A typical score for a refined structure lies in between -3 and -1 REU per AA [12], [31].
• *Delta isoelectric point:* The isoelectric point (pI) is the pH at which the protein carries no net charge. At lower pH levels, the protein tends to be positively charged, and negatively charged at higher ones. We show the difference in pI to the corresponding template protein.
• *aggrescan3d max:* Aggregation of proteins is a commonly faced issue which is often caused by the exposure of hydrophobic regions on the protein's surface. An aggregation score is calculated for each residue by aggrescan3d [32]. We report its maximum value over all residues in the protein. Lower values mean less risk of aggregation.

### 4.5 Alternative genotype selection

The *Results* section centers on tailoring the design process to a single patient's MHC-I genotype (the primary genotype). However, we also demonstrate CAPE-Beam's ability to generalize to other genotypes by comparing its performance on the primary- and an alternative genotype. To construct this alternative genotype, we began by randomly generating $3\times 10^{6}$ 8-10mers and predicting their presentation ranks across more than 250 of the most common MHC-I alleles using NetMHCpan. Peptides with a predicted rank below 2% were considered presented - otherwise hidden. We then defined the distance between two MHC-I alleles as the percentage of the peptides where their predicted presentation status differed. For each MHC-I gene in the alternative genotype (HLA-A, HLA-B and HLA-C) we selected the two alleles that maximized the minimum distance to any alleles in the primary genotype – i.e., the alleles that are most dissimilar in presentation profile.

## 5 Results and discussion

In this section, we explore the ability of the CAPE-Beam decoding strategy described in Section 4.2 to design proteins that exhibit reduced visibility to CTLs, while maintaining structural fidelity. Reduced CTL visibility is achieved by increasing similarity to human proteins and minimizing predicted presentation via the MHC-I pathway.

This analysis is based on a hypothetical patient with the following MHC-I genotype: HLA-A*02:01, HLA-A*24:02, HLA-B*07:02, HLA-B*39:01, HLA-C*07:01, and HLA-C*16:01. To demonstrate the generalizability of CAPE-Beam to other genotypes, the approach's performance on this genotype and an alternative one are compared at the end of the section. The protein templates used for design are those described in Section 4.3.

We introduce a naming convention to be able to easily refer to different CAPE-Beam configurations. Each variant is denoted by CB, followed by its min_self_kmer_len and its non_self_prob_factor. For instance, the variant that enforces self-5mers and uses a non_self_prob_factor of 0.9 will be referred to as CB 5mers (0.9).

**Fig. 2:**
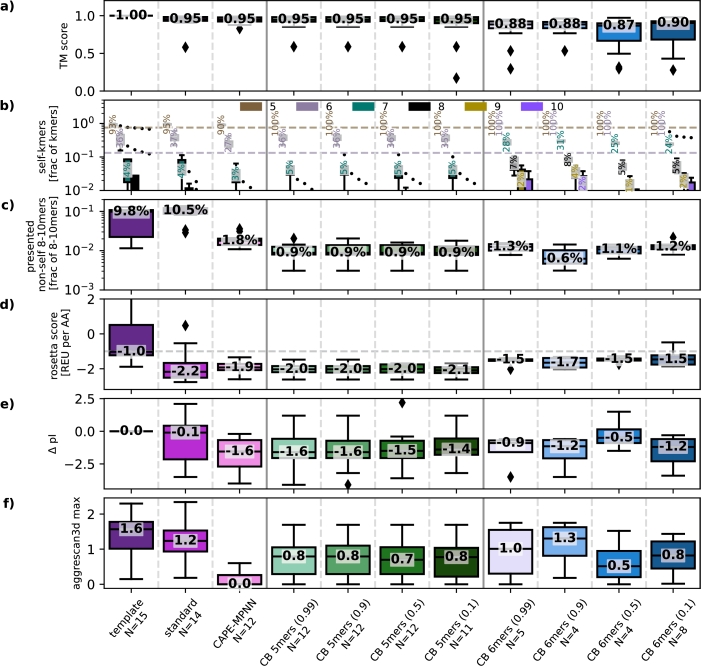
**Structural similarity, CTL reaction risk and molecular properties (validation set):** These boxplots show metrics based on the PDB validation set template structures as well as the corresponding predicted structures of generated designs (see Section 4.4.1). The numbers on the plots show the median values. Except for **a)**, all the charts only consider designs that have a TM-score above 0.9. The number of qualifying designs per source can be found as *N* under its name. **a)** shows the TM-score between predicted structures and the corresponding template proteins. We see that enforcing longer self-kmers or (less so) applying stronger penalties for non-self kmers (i.e., lower non_self_prob_factor) tends to reduce structural fidelity. **b)** Only for non-human proteins, this plot shows the proportion of 5-10mers found in the human genome. The dashed background lines represent expected frequencies if kmers were uniformly sampled from standard AAs. While ProteinMPNN or CAPE-MPNN designs introduce a significant number of non-self 5mers, CAPE-Beam was able to enforce self-5mers in most designs while still achieving predicted TM-scores above 0.9. In many cases, CAPE-Beam succeeds in meeting this criterion even when enforcing self-6mers. **c)** Presented non-self peptides will potentially be immunogenic. CAPE-Beam consistently generates the fewest such peptides. With regards to stability **d)**, the best designs seem to be generated by the standard way of sampling from ProteinMPNN. However, most of the considered designs were well within the typical range for refined structures. Plot **e)** shows that both deimmunization methods produced designs that tend to have lower pIs than their templates. With regards to the aggregation scores **f)** all designs seem to be in the range or better than the original templates.

Based on the validation templates, Fig. 2 compares the designs generated using various CAPE-Beam decoding strategy hyper-parameters to the benchmarks (template, standard, CAPE-MPNN), with respect to structural similarity (Fig. 2a), CTL reaction risk (Fig. 2b,c), and some predicted molecular properties (Fig. 2d-f, see Section 4.4). We evaluated the hyperparameter combinations in Table 1. Only sources which produced at least one design with a TM-score above 0.9 are shown in the figure.

We observe that enforcing longer self kmers reduces the quality of the generated designs (Fig. 2a). Requiring all 7mers or longer to originate from the human proteome results in suboptimal designs, highlighting the challenge of achieving effective de-immunization with respect to 8-10mer peptides within the restricted sequence space of the human proteome. Nevertheless, for many templates CAPE-Beam is able to produce high-quality designs even when all 6mers are constraint to come from the human proteome, which increases the mimicry to the human proteome as shown in Fig. 4.

The relationship between the non_self_prob_factor and design quality appears to be less straightforward. As the penalty for incorporating hidden non-self kmer continuations is increased (i.e., by lowering the non_self_prob_factor), we initially observe only modest changes in median TM-score, although accompanied by a broader distribution. However, when the penalty becomes absolute (non_self_prob_factor = 0), no designs meet the TM-score threshold anymore. We hypothesize that ProteinMPNN has strong preferences for specific sequence continuations, and that only substantial penalization is capable of diverting the model from its favored generation path.

Fig. 2b then depicts the percentage of self-kmers in the protein sequence. It only considers templates that do not originate from the human proteome. The three benchmarks only substantially incorporated self-kmers up to length 6. In contrast, CAPE-Beam can be configured to enforce longer self-kmers. Importantly, enforcing self 5mers still allows for high predicted TM-scores, which is noteworthy since the benchmarks already include many non-self 5mers. In cases where enforcing self 6mers still resulted in acceptable TM-scores, this also led to the inclusion of more self 7–10mers. These longer self-kmers further enhance mimicry of the human proteome, as illustrated in Fig. 4.

The third boxplot Fig. 2c presents the distribution of predicted presented non-self 8-10mers (potentially immunogenic). In principle, all CAPE-Beam designs should contain zero of these, since the decoding strategy is explicitly designed to avoid them. However, due to the use of a faster, approximate antigen presentation predictor during design (as explained in Section 4.1), this is not always the case in practice. When we re-evaluated the designs using the same predictor that was used during decoding, no non-self presented 8-10mers were detected in CAPE-Beam designs, confirming that the discrepancy arises from differences between the prediction tools. In the future, fast and accurate MHC-I presentation predictors could allow the same predictor to be used consistently during both – design and evaluation. Even with the current setup, CAPE-Beam designs still contain significantly fewer potentially immunogenic kmers than the benchmarks.

The final three boxplots Fig. 2d-f compare some molecular properties of generated sequences. ProteinMPNN was trained to find the optimal sequence for a given spatial backbone template. Theoretically, adding constraints to any optimization problem can only degrade performance with respect to this original objective - which was highlighted in Fig. 2a. However, the constraint might naturally also cause biases with regards to other properties as well. Avoiding antigen presentation is such a constraint. So, although our main objective is to create less immunogenic proteins that retain high structural similarity to their template, we included Fig. 2d-f to demonstrate how guiding ProteinMPNN toward reduced immunogenicity might affect other commonly considered metrics. Since every therapeutic will have different other constraints to fulfill, these only have an informative character. With regards to the Rosetta score per AA (Fig. 2d), the template structures themselves often exhibit values above the normal -1 REU per AA. This might be because they were taken directly from PDB entries without the relaxation step applied during structure prediction by tools like AlphaFold-2. In contrast, nearly all generated designs with TM-scores above 0.9 fall within an acceptable Rosetta score range. Regarding pI values (Fig. 2e), both fine-tuning via CAPE-MPNN and guided decoding via CAPE-Beam tend to result in designs with lower pI than the original proteins. This observation may warrant further investigation to understand its cause and potential implications. Finally, in terms of aggregation scores (Fig. 2f), the designed sequences are comparable to or even better than the original proteins.

**Fig. 3:**
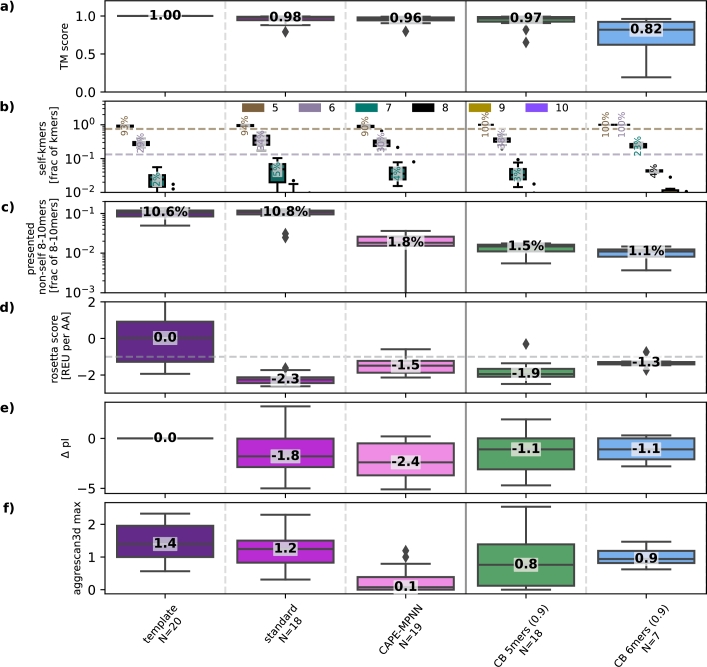
**Structural similarity, CTL reaction risk and molecular properties (test set):** This figure shows the same information as Fig. 2 - only for the test set.

**Fig. 4:**
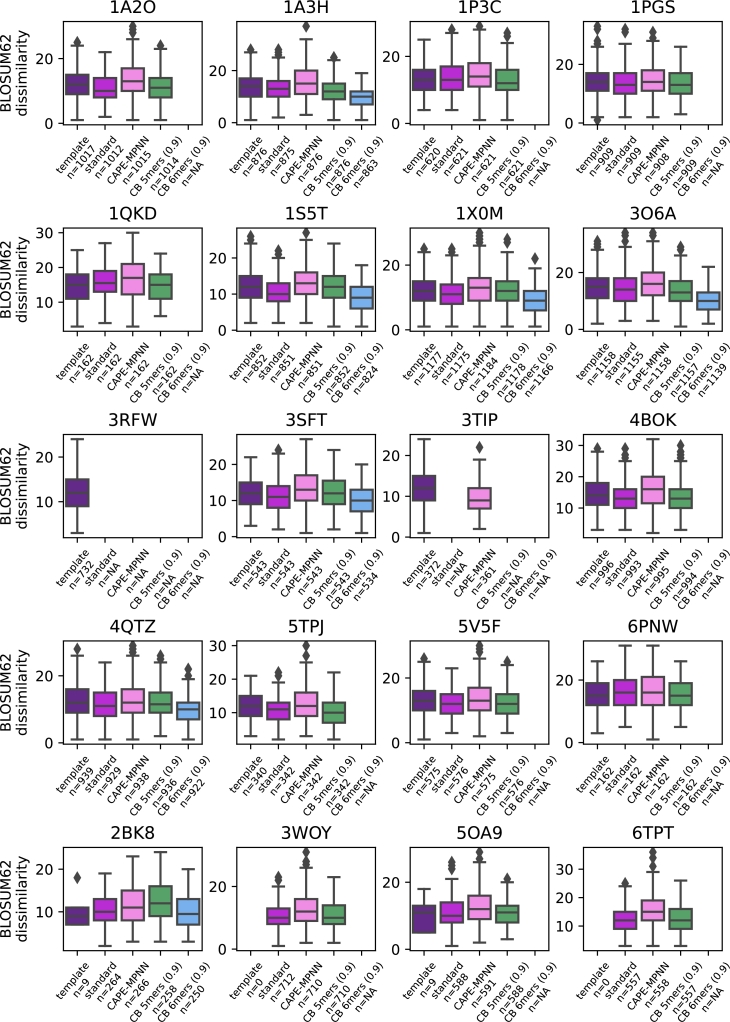
**CB 6mers (0.9) generated 8-10mers most similar to the self-proteome for non-human proteins in the test set:** Dissimilarity to the human proteome has been shown to be linked to immunogenicity [7], [8], [9]. This figure shows the distribution of our BLOSUM62 dissimilarity scores (see Section 4.4.2) for non-self 8-10mers in the 20 test set proteins. The number *n* shown below each source label indicates how many 8-10mers are non-self. Only designs with a predicted TM-score above 0.9 are included; sources without any such designs are marked with *n* = *NA*. We find that enforcing all 6mers to come from the human proteome results in the lowest dissimilarity scores for non-human proteins — indicating increased mimicry of self-peptides. An exception is observed for human proteins or their homologs (e.g., 2BK8, 3WOY, 5OA9, 6TPT), where the original templates naturally contain few non-self kmers, and those that exist tend to be more similar by definition. Still, CAPE-Beam 6mers consistently generate less dissimilar kmers than both the 5mer constraint and the standard decoding approach.

Based on the TM-score distributions observed in Fig. 2a, which showed more variability and less reliable generation at lower non_self_prob_factor values, we selected CB 5mers (0.9) and CB 6mers (0.9) for further evaluation on the test-set templates in Fig. 3, Fig. 4. In Fig. 3, we observe a similar picture as seen in the validation set (Fig. 2). However, the performance of CB 6mers (0.9) is slightly worse in terms of structural similarity. Nevertheless, the designs that surpass the predicted TM-score threshold still show comparable kmer composition (Fig. 3b, c) and similarly good molecular properties (Fig. 3d-f). Interestingly, for the test set templates, there is little difference in pI between the CAPE-Beam designs and those produced by standard sampling.

Not every presented non-self peptide will trigger an immune response. Next to other factors, the similarity of a peptide to the human proteome is known to influence its immunogenicity [7], [8], [9]. Fig. 4 investigates the hypothesis that CAPE-Beam tends to incorporate 8-10mers that are more similar to the human proteome. Overall, we find that this is true for CB 6mers (0.9) compared to all benchmarks - except for the template itself, if it is derived from the human proteome or a homolog.

**Fig. 5:**
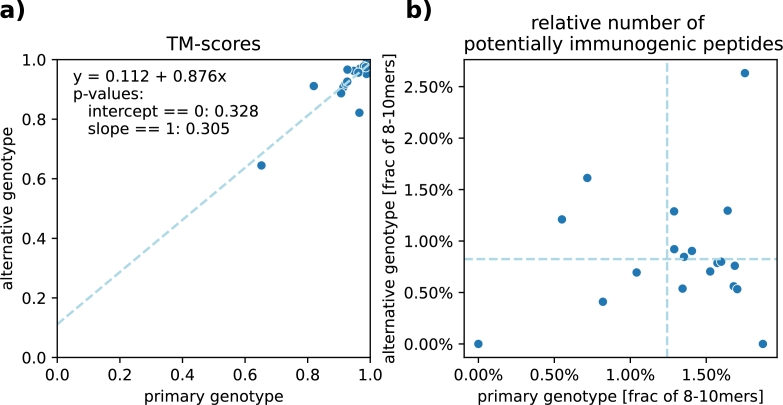
**Comparing structural similarity and reduction of potentially immunogenic peptides in CAPE-Beam designs across two MHC-I genotypes:** Every dot in this figure represents two designs generated for the same test set template – one using the primary genotype and one using the alternative genotype. The x-axis displays the metric's value for the primary genotype design, while the y-axis shows the value for the alternative genotype design. **a)** compares the predicted TM-scores (see Fig. 3a). The strong correlation indicates that structural quality is largely preserved across genotypes. The dotted line shows the linear regression. **b)** compares the fraction of presented non-self 8-10mers (see Fig. 3c). The two dotted lines show the mean values. We observe that this fraction is similarly reduced for both genotypes, suggesting that CAPE-Beam effectively lowers immunogenic risk regardless of genotype.

*Other genotypes*  MHC-I genes are highly polymorphic. To test whether our method can also be applied to genotypes beyond the primary genotype used throughout this section, we also designed sequences for the test templates assuming an alternative genotype: HLA-A*29:02, HLA-A*30:07, HLA-B*15:13, HLA-B*57:01, HLA-C*14:02 and HLA-C*04:04. This genotype was chosen to be as different as possible from the primary one (see Section 4.5). Fig. 5a demonstrates that CAPE-Beam produces sequences with equally high predicted structural similarity under both genotypes. Furthermore, the number of potentially immunogenic kmers was also reduced to a comparable extend (Fig. 5b). It is important to note again that when using the same presentation predictor during evaluation as was used during decoding, these values are all zero. These results support the conclusion that our method can be effectively applied to other MHC-I genotypes.

*Limitations*  Although we have tested the generalizability of CAPE-Beam to other MHC-I alleles in the paragraph above, there might still be MHC-I genotypes for which certain proteins cannot be deimmunized. Also, to achieve broader population coverage of produced therapeutics, future work should test avoiding a set of commonly presented kmers while on the other hand extending the length of incorporated self kmers. As the number of 3D structures in the PDB increases and explores more of the landscape of potential proteins, the foundation models' understanding of the sequence-structure relationship will improve. This might also improve their ability to find alternative paths through the sequence generation tree.

Future work should also include laboratory confirmation of the designs, instead of relying solely on bioinformatics methods. In particular, structural accuracy could be confirmed using X-Ray crystallography. Parallel experiments could induce the expression of both the native and designed proteins from mRNA constructs in mouse models humanized for MHC-I genes. Immune responses could then be measured using T-cell proliferation assays and ELISpot. If a kmer is detected to be immunogenic in these experiments, CAPE-Beam can easily be adjusted to avoid this in the next iteration.

Another limitation of our work is, that we focus on the canonical MHC-I kmer lengths of 8–10 AAs. Although some immunogenic peptides may be longer, many of these will still contain a 8–10 AA long core sequence, which is effectively covered by our method.

## 6 Conclusion

With CAPE-Beam, we present a decoding strategy for ProteinMPNN that allows to control immunogenicity risk in protein design. It enables the generation of protein sequences composed entirely of segments up to a length 10 that are (1) either predicted not to be presented to CTLs (hidden) or are (2) found in the human proteome (self-kmers) and thus subject to central tolerance. Moreover, for certain proteins, we showed that it is possible to increase the minimum required self-kmer length to 6 – increasing the mimicry to the human proteome. While we were not able to extend this constraint to cover all peptide lengths presented by MHC-I, this should still be beneficial in light of results that show that a peptide's similarity to the human proteome is linked to lower immunogenicity risk [7], [8], [9]. Finally, we compared our method to a fine-tuning approach (CAPE-MPNN) and found that CAPE-Beam has higher potential for applications in precision medicine.

A strength of our method is that it can assure the user that all non-self 8-10mers in the designed sequences are predicted not to be presented via the MHC-I pathway. This ability provides a strong immunogenicity safeguard. Therefore, CAPE-Beam represents an important step towards using generative artificial intelligence in developing protein therapeutics that are more safe when tailored to individual patients. Future extensions to our methodology – increasing self k-mers to cover all MHC-I presented lengths – are a promising path towards reducing immunogenicity across populations.

## CRediT authorship contribution statement

**Hans-Christof Gasser:** Writing – review & editing, Writing – original draft, Visualization, Validation, Software, Methodology, Conceptualization. **Ajitha Rajan:** Writing – review & editing, Visualization, Supervision, Conceptualization. **Javier A. Alfaro:** Writing – review & editing, Visualization, Supervision, Conceptualization.

## Declaration of Competing Interest

The authors declare that they have no known competing financial interests or personal relationships that could have appeared to influence the work reported in this paper.

## Figures and Tables

**Table A.3:** Log-PWM for presented 9-mers by HLA-A*02:01: Each column corresponds to a position in the 9-mer and each row to one of the 20 standard AAs. The values are the log-probabilities of finding the corresponding AA at that position in a presented 9-mer.

	position								
	1	2	3	4	5	6	7	8	9
A	-2.72	-3.73	-2.90	-3.02	-3.19	-3.24	-3.14	-3.00	-2.24
C	-4.10	-5.25	-3.94	-3.90	-3.66	-3.72	-3.72	-3.51	-3.28
D	-5.33	-8.24	-2.46	-2.23	-2.97	-3.23	-3.61	-4.01	-inf
E	-4.37	-6.63	-3.87	-2.18	-2.95	-3.10	-3.10	-2.62	-6.96
F	-2.20	-4.79	-2.42	-3.51	-2.88	-2.92	-2.69	-2.87	-3.57
G	-2.88	-5.96	-3.53	-2.67	-2.84	-3.35	-4.47	-3.13	-7.05
H	-3.00	-8.79	-3.25	-3.09	-2.78	-3.22	-2.52	-2.78	-7.70
I	-2.85	-2.12	-2.76	-3.66	-2.81	-2.36	-2.70	-3.64	-1.92
K	-2.56	-7.24	-3.90	-3.03	-2.89	-3.39	-3.77	-2.86	-7.41
L	-3.10	-1.14	-2.73	-3.49	-3.05	-2.55	-2.82	-3.00	-1.51
M	-2.89	-1.44	-2.51	-3.63	-3.30	-2.89	-2.87	-3.08	-2.33
N	-3.38	-7.88	-3.05	-2.92	-3.12	-3.02	-3.20	-3.03	-inf
P	-5.60	-7.70	-3.18	-2.30	-2.78	-2.59	-2.55	-3.31	-5.26
Q	-3.30	-2.13	-3.30	-2.98	-3.03	-2.90	-2.94	-2.60	-7.70
R	-2.80	-7.98	-4.59	-3.45	-3.21	-3.73	-3.68	-2.97	-6.57
S	-2.72	-4.24	-3.12	-2.81	-3.15	-2.98	-3.31	-2.74	-4.79
T	-3.05	-3.06	-3.49	-3.11	-3.06	-2.84	-2.93	-2.62	-3.43
V	-2.94	-2.36	-3.24	-3.60	-2.92	-2.52	-2.72	-3.39	-1.19
W	-3.38	-6.05	-2.35	-3.33	-2.79	-3.48	-2.52	-3.43	-5.67
Y	-2.20	-6.23	-2.44	-3.47	-2.92	-3.26	-2.79	-2.61	-5.51

**Table A.4:** **Confusion matrix:** TP, FP, FN and TN denote the number of examples categorized based on the joint predictions from PWM predictor and NetMHCpan as defined by the confusion matrix.

PWM ﹨ NetMHCpan	Presented	Not Presented
Presented	True positives (TPs)	False positives (FPs)
Not Presented	False negatives (FNs)	True negatives (TNs)

**Table A.5:** **Replication of NetMHCpan-4.1 predictions by the PWM classifier for the primary genotype:** Assessed on 3 × 10^6^ train and 3 × 10^5^ test peptides. The way the threshold is calibrated balances precision and recall.

Allele	kmer len	Train peptides			Test peptides		
		accuracy	precision	recall	accuracy	precision	recall
HLA-A*02:01	8+9+10	0.995	0.810	0.809	0.995	0.801	0.799
HLA-A*02:01	8	0.999	0.736	0.732	0.999	0.758	0.771
HLA-A*02:01	9	0.993	0.864	0.863	0.992	0.854	0.857
HLA-A*02:01	10	0.993	0.690	0.689	0.993	0.677	0.664

HLA-A*24:02	8+9+10	0.987	0.845	0.844	0.986	0.842	0.840
HLA-A*24:02	8	0.993	0.807	0.806	0.993	0.810	0.808
HLA-A*24:02	9	0.983	0.874	0.874	0.982	0.873	0.864
HLA-A*24:02	10	0.984	0.815	0.815	0.984	0.808	0.814

HLA-B*07:02	8+9+10	0.994	0.820	0.818	0.993	0.826	0.800
HLA-B*07:02	8	0.996	0.780	0.778	0.996	0.785	0.762
HLA-B*07:02	9	0.993	0.855	0.854	0.992	0.856	0.839
HLA-B*07:02	10	0.993	0.791	0.790	0.992	0.805	0.768

HLA-B*39:01	8+9+10	0.990	0.827	0.827	0.990	0.830	0.829
HLA-B*39:01	8	0.994	0.819	0.817	0.994	0.819	0.818
HLA-B*39:01	9	0.983	0.853	0.853	0.984	0.858	0.856
HLA-B*39:01	10	0.991	0.755	0.754	0.991	0.755	0.755

HLA-C*07:01	8+9+10	0.988	0.836	0.835	0.988	0.838	0.839
HLA-C*07:01	8	0.995	0.808	0.806	0.996	0.820	0.806
HLA-C*07:01	9	0.976	0.855	0.854	0.977	0.860	0.861
HLA-C*07:01	10	0.993	0.755	0.749	0.992	0.728	0.740

HLA-C*16:01	8+9+10	0.993	0.852	0.851	0.993	0.855	0.851
HLA-C*16:01	8	0.994	0.846	0.844	0.994	0.843	0.841
HLA-C*16:01	9	0.988	0.871	0.871	0.988	0.877	0.873
HLA-C*16:01	10	0.997	0.720	0.719	0.996	0.725	0.717

**Table A.6:** **Replication of NetMHCpan-4.1 predictions by the PWM classifier for the alternative genotype:** Assessed on 3 × 10^6^ train and 3 × 10^5^ test peptides. The way the threshold is calibrated balances precision and recall.

Allele	kmer len	Train peptides			Test peptides		
		accuracy	precision	recall	accuracy	precision	recall
HLA-A*29:02	8+9+10	0.990	0.867	0.867	0.990	0.866	0.866
HLA-A*29:02	8	0.996	0.856	0.855	0.996	0.855	0.848
HLA-A*29:02	9	0.983	0.876	0.875	0.983	0.871	0.873
HLA-A*29:02	10	0.989	0.855	0.855	0.990	0.859	0.860

HLA-A*30:07	8+9+10	0.990	0.860	0.860	0.990	0.865	0.860
HLA-A*30:07	8	0.996	0.842	0.842	0.996	0.846	0.821
HLA-A*30:07	9	0.983	0.869	0.869	0.984	0.871	0.872
HLA-A*30:07	10	0.990	0.851	0.851	0.990	0.861	0.853

HLA-B*15:13	8+9+10	0.986	0.852	0.852	0.986	0.851	0.853
HLA-B*15:13	8	0.994	0.817	0.817	0.993	0.809	0.810
HLA-B*15:13	9	0.979	0.872	0.872	0.979	0.872	0.877
HLA-B*15:13	10	0.986	0.824	0.824	0.986	0.826	0.823

HLA-B*57:01	8+9+10	0.988	0.850	0.849	0.988	0.849	0.844
HLA-B*57:01	8	0.993	0.829	0.829	0.992	0.829	0.810
HLA-B*57:01	9	0.988	0.882	0.880	0.988	0.880	0.880
HLA-B*57:01	10	0.983	0.825	0.824	0.983	0.826	0.820

HLA-C*14:02	8+9+10	0.988	0.855	0.854	0.988	0.855	0.855
HLA-C*14:02	8	0.990	0.842	0.842	0.989	0.839	0.838
HLA-C*14:02	9	0.983	0.879	0.877	0.982	0.879	0.878
HLA-C*14:02	10	0.991	0.796	0.795	0.991	0.799	0.804

HLA-C*04:04	8+9+10	0.990	0.840	0.839	0.990	0.846	0.841
HLA-C*04:04	8	0.994	0.823	0.823	0.995	0.842	0.829
HLA-C*04:04	9	0.980	0.855	0.854	0.980	0.857	0.854
HLA-C*04:04	10	0.996	0.724	0.720	0.997	0.743	0.737

## Appendix A PWM MHC-I presentation predictor

This predictor follows a common position-specific scoring matrix (PSSM) approach for motif finding to identify peptides presented on the cell's surface by MHC-I variants. For each MHC-I allele and each kmer length 8-10 a separate PWM is used. The process of generating those is described below for one particular kmer length (9) and one particular MHC-I allele (HLA-A*02:01). First one million independent and identically distributed (iid) random 9mers are sampled. At each position in a kmer we independently sample from a uniform distribution over all standard AAs. Then NetMHCpan-4.1 is used to identify the presented kmers using a rank cut-off of 2%. These predicted to be presented kmers are used to create the PWM.

Each row of the PWM represents one AA type and each column one position in the peptide. The values are the probabilities of finding the AA at that position in the presented kmers. For example, the probability of finding a methionine (row 11) at position two (column 2) of a 9-mer is the percentage of predicted to be presented 9-mers that have a methionine at position two (23.8%, see $\exp({\text{PWM}}_{11,2})$ in Table A.3). Typically, these probabilities would be adjusted by the background probability to arrive at score values. Since we have sampled from a uniform distribution this adjustment would have no influence on the final classification and we can simply directly calculate the PSSM by taking the log of the PWM at each position. For HLA-A*02:01 9mers the resulting score matrix is displayed in Table A.3:

To assess the presentation of a new peptide, we calculate the sum of its AAs' log-probabilities above and compare it to a pre-calculated threshold. If it is higher than that threshold, the peptide is predicted to be presented – otherwise not. The threshold is determined to ensure that the same percentage *p* of random peptides are predicted to be presented by NetMHCpan-4.1 as by the PWM predictor (e.g. 2.6% for 9mers presented by HLA-A*02:01). Then the sum of log-probabilities for each 9-mer of the random peptides needs to be calculated using the log-PWM. The threshold is then the *p*-percentile of these log-probabilities. In our case this is -24.84.

Let us classify the peptide *SMELDKVEI*. The log-probabilities for finding its AAs at their respective positions in NetMHCpan predicted to be presented peptides are: -2.72, -1.44, -3.87, -3.49, -2.97, -3.39, -2.72, -2.62 and -1.92. Their sum is -25.13 which is lower than the log-probability threshold of -24.84 we calculated above. We, therefore, predict that the peptide is not presented. If in contrast the terminal position had been a valine, which was observed in 30.3% of all NetMHCpan predicted to be presented peptides, then the sum of the log-probabilities would have been -24.41, which would be above the threshold and therefore the PWM predictor would predict the peptide to be presented.

Another question is how similar the predictions of the PWM based predictor are to those of NetMHCpan. The following Table A.5, Table A.6 address this by displaying the accuracy, precision and recall on the alleles of the two MHC-I genotypes considered in the paper, based on the following Formulas (A.1) and Table A.4. We calculated these metrics for the peptides used to generate the PWMs (train) as well as for a smaller independent set (test).

(A.1) $$\text{Accuracy}=\frac{TP+TN}{TP+TN+FP+FN}\;\text{Precision}=\frac{TP}{TP+FP}\;\text{Recall}=\frac{TP}{TP+FN}$$

Since the problem of MHC-I presentation prediction is highly unbalanced (only around 2 in 100 peptides will be presented by a single MHC-I) variant, accuracy is not a good measure of performance. We, therefore, look at the precision and recall of the PWM classifier. Table A.5, Table A.6 show that the way we selected the threshold, balances these two metrics. We can also observe the best performance for 9mers, while performance deteriorates slightly for other lengths. That precision and recall both typically exceed 80%, gives us confidence that the predictor will be able to guide our method down reasonable paths. However, CAPE-Beam would still benefit from using NetMHCpan directly during decoding - although this is computationally more expensive.

## Footnotes

2 https://seq2fun.dcmb.med.umich.edu/TM-align/.
